# A robust approach for endotracheal tube localization in chest radiographs

**DOI:** 10.3389/frai.2023.1181812

**Published:** 2023-05-12

**Authors:** Chung-Chian Hsu, Rasoul Ameri, Chih-Wen Lin, Jia-Shiang He, Meghdad Biyari, Atefeh Yarahmadi, Shahab S. Band, Tin-Kwang Lin, Wen-Lin Fan

**Affiliations:** ^1^Department of Information Management, International Graduate School of Artificial Intelligence, National Yunlin University of Science and Technology, Douliu, Taiwan; ^2^Department of Information Management, National Yunlin University of Science and Technology, Douliu, Taiwan; ^3^Buddhist Dalin Tzu Chi Hospital, Chiayi, Taiwan; ^4^School of Medicine, Tzu Chi University, Hualien, Taiwan; ^5^Future Technology Research Center, National Yunlin University of Science and Technology, Douliu, Taiwan; ^6^International Graduate School of Artificial Intelligence, National Yunlin University of Science and Technology, Douliu, Taiwan

**Keywords:** endotracheal tube, chest radiograph, deep learning, medical image segmentation, U-Net++

## Abstract

Precise detection and localization of the Endotracheal tube (ETT) is essential for patients receiving chest radiographs. A robust deep learning model based on U-Net++ architecture is presented for accurate segmentation and localization of the ETT. Different types of loss functions related to distribution and region-based loss functions are evaluated in this paper. Then, various integrations of distribution and region-based loss functions (compound loss function) have been applied to obtain the best intersection over union (IOU) for ETT segmentation. The main purpose of the presented study is to maximize IOU for ETT segmentation, and also minimize the error range that needs to be considered during calculation of distance between the real and predicted ETT by obtaining the best integration of the distribution and region loss functions (compound loss function) for training the U-Net++ model. We analyzed the performance of our model using chest radiograph from the Dalin Tzu Chi Hospital in Taiwan. The results of applying the integration of distribution-based and region-based loss functions on the Dalin Tzu Chi Hospital dataset show enhanced segmentation performance compared to other single loss functions. Moreover, according to the obtained results, the combination of Matthews Correlation Coefficient (MCC) and Tversky loss functions, which is a hybrid loss function, has shown the best performance on ETT segmentation based on its ground truth with an IOU value of 0.8683.

## Introduction

ETT is a wide-bore plastic tube that is inserted into the trachea to provide the capability of artificial ventilation for patients with in intensive care units (ICU). These tubes are produced in different sizes and have a balloon at the tip to prevent gastric contents entering into the lungs. Adult tubes are usually 1 cm in diameter. These tubes are visible on radiographs since a radiopaque strip is designed within it (Chethan and Hughes, 2008).

Computed Tomography (CT) and X-ray imaging techniques are widely used in medical applications, such as for the classification of COVID-19 (Barstugan et al., 2020; Özkaya et al., 2020; Öztürk et al., 2021) and the diagnosis of tumors (Aswathy and Kumar, 2023). A frontal chest radiograph is usually used to assess the ETT position. The coordinate of the ETT in the patient's body also relies on the position of his or her head; if the neck is flexed, the tip of the tube would be in the trachea (Henschke et al., 1997). Chest radiography has been used to confirm the ETT position in ICU but, in most cases, this method causes delays which could lead to serious complications and even death. Considering these facts, experts have advised having a precise ETT placement which needs to be confirmed through clinical signs and the detection of exhaled carbon dioxide. The urgency of improving traditional ETT detection methods has been raised and researchers have focused on the implementation of machine vision and image processing techniques to obtain a precise and fast detection. In fact, accuracy has played a key role in models; thus, this paper has tried to improve this criterion through applying different loss functions.

In order to achieve proper positioning of the inserted ETT, the American college of radiology recommends the acquisition of chest radiographs during intubations in the ICU (Godoy et al., 2012). Their findings showed that, in about 15% of cases, a repositioning of ETT was required (Brown et al., 2022). The safe distance of an inserted ETT within the mid trachea is about 7 cm above the carina as its upper boundary and at least 3 cm away from this part of the patient's body as its lowest boundary (Brown et al., 2022).

It is worth mentioning that there might be a risk of incorrect intubation which may lead to vocal cord injury if the mentioned restrictions are neglected (Popat et al., 2012). If the ETT tip gets too close to the carina, situations such as the partial or complete collapse of the lung, hyperinflation of the lung, pneumothorax, or even death of the patient could occur.

Various suggestions have been proposed so far, such performing radiography after ETT intubation. The ICU is usually equipped with a portable X-ray machine and chest radiographs (CXRs) are obtained in a supine anteroposterior (AP) view, however several challenges, such as external monitoring devices, tubes, or catheters, can cause ambiguity in the position of an ETT and carina which can restrict the detection accuracy (Mao et al., 2022). Thus, in order to reduce these complications, different methods to obtain an accurate ETT detection have been the main concern of researchers in this area. Increasingly they have focused on computer-aided detection (CAD) methods to enhance the detection of incorrect ETT intubation and reduce the burden on healthcare systems.

The applied strategy in previous automatic CAD methods for ETT detection can be summarized in four steps: preprocessing, finding the neck, seed generation, and region growing (Mao et al., 2022). Having applied feature extraction to detect ETT, these methods obtained reasonable outcomes. However, the mentioned approaches are not able to solve the problem since the manual templates and hyperparameters should be determined according to experience or the implemented morphology in the process (Goodman et al., 1976).

An artificial neural network, which is a type of advanced machine learning algorithm, can be considered as the basis for the most important deep learning techniques. More recently, different deep learning techniques have been applied to a wide range of vital demands related to image processing, including image segmentation, which has played an important role in this research to detect the ETT (Goodman et al., 1976).

Detection of the tip position of the ETT using X-ray images in order to reduce the related error to the ground truth value is the main concern of the presented study. Although deep learning models are widely used for ETT segmentation, finding appropriate loss functions and encoders has remained a major challenge. Because of this, finding the best loss function is one of the main objectives of the presented paper. For this purpose, different loss functions from distribution and region-based groups are evaluated in this study. Then, their different integrations are used to obtain the best compound loss function for ETT segmentation and localization. Moreover, three well-known encoders are studied to obtain the best feature extraction network to achieve the predefined purpose.

Efforts have been made to implement compound loss functions based on distribution and region loss functions to enhance the results for ETT segmentation and localization in this paper. A U-net++ model has been optimized based on different loss functions and different encoders. Finally, its performance has been evaluated by implementing different loss functions.

Our paper makes several significant contributions to the field of ETT segmentation and localization. Firstly, we collected expertly annotated data from the Dalin Tzu Chi Hospital, ensuring high-quality data for accurate ETT segmentation. Secondly, we proposed novel compound loss functions based on distribution and region-based approaches, designed to address specific challenges in ETT segmentation and improve accuracy. We evaluated the effectiveness of various loss functions and combined them to train the U-Net++ model. Thirdly, we investigated the impact of different encoders on ETT segmentation performance, focusing on RegNet and ResNet encoders with U-Net++ architecture. This exploration was important to identify limitations in existing encoders and provide insights into selecting the most effective ones for this task. Our experimental results demonstrated that the proposed combination of distribution and region-based loss functions outperformed single loss functions for ETT segmentation and localization.

The paper is structured as follows. In Section 2, we provide a brief review of previous works on ETT segmentation and localization. Section 3 outlines our proposed methods based on U-Net++ and introduces two main categories of loss function. The results and discussion are presented in Section 4, and the conclusion is provided in Section 5.

## Related works

Portability, rapid image acquisition, and availability of immediate information on the bedside have turned chest radiography into the most common imaging technique in the ICU. The severity of underlying disease and the frequency with which a patient needs to be monitored are the two main criteria making ICU patients more likely to develop side effects of their disease or intervention. To monitor the disease process and also prevent further difficulties from interventions, the existence of a portable chest radiography in the ICU is essential; however, it is subject to overuse especially in patients with a stable condition. Chest radiograph usage in the ICU should not be used when it could lead to severe harm. The emerging role of bedside lung ultrasound, which was implemented by clinicians, has been studied in recent literature (Suh et al., 2015).

Detection of an endotracheal tube (ETT) in the patient's body with high precision is vital in the ICU setting, where timely identification of a mispositioned support device like an ETT may prevent mortality. Thus, a series of deep learning-based algorithms which have been designed to detect ETT's position relative to the carina on chest radiographs has been proposed (Kara et al., 2021). In some cases, patients with ETT intubation are most likely to receive a chest x-ray (CXR) to see if the process has been correctly completed or if it should be repositioned. A radiologist evaluates the ETT position with its ground truth value (Harris et al., 2021). A machine learning model, such as the updated version of the YOLO-V3 framework, is applied to achieve this objective. To measure the ETT-Carina distance, a V3 deep Neural Network has been developed and showed its effective performance for detecting ETTs. The efficiency of deep learning segmentation models for ETT position on frontal CXRs has been studied in Schultheis and Lakhani (2022).

Deep learning has demonstrated an acceptable performance on the images and the obtained outcomes of CNN models can be applied on the image datasets, which have been designed for feature extraction from images.

Previously, CNN models were not capable of dealing with temporal or spatial features. This motivated researchers to create “U-Net” models, which contain two sections: the first part is a “classic” Convolutional Neural Network that scans the image, extracts its related patterns, then adds high resolutions features to them. Then, to recreate a full binary image, the predesigned network increases the scale of its hidden layers. Taking a full image as input and generating another image as the output can be considered as the expected task of the presented models. It is expected that, in the output image, the background would be delimited from the existing object since it contains values of 0 and 1 (Du et al., 2020).

Later U-net++ models have implemented dense block ideas to improve the system three ways, namely having convolution layers on skip pathways, having dense skip connections on skip pathways and having deep supervision, which enables model pruning (Zhou et al., 2019).

The efficacy of deep convolutional neural networks (DCNNs) in determining subtle, intermediate, and more obvious image differences in radiography has been studied in Lakhani (2017). Others have investigated different aspects, such as if a directed clinical evaluation could eliminate the need for routine daily CXRs (Brengman et al., 1999). Other studies like Brengman et al. (1999) believes that on demand radiography is equivalent to daily routine chest radiography (Brengman et al., 1999). The frequency of daily CXRs for patients who were hospitalized in the USA had been monitored in Hejblum et al. (2008).

In this paper, U-Net++ model is used for ETT segmentation. Different well-known encoders and loss functions are evaluated to obtain the best-trained U-Net++. Then, the compound loss functions based on distribution and region-based loss functions are proposed to achieve the best results for ETT segmentation and localization.

## Methodology

Deep learning structures were used to obtain the best performance for ETT segmentation (Goodman et al., 1976). According to the literature and our research, selecting appropriate encoders and loss functions has an effective impact on ETT segmentation and localization.

**Figure 1:**
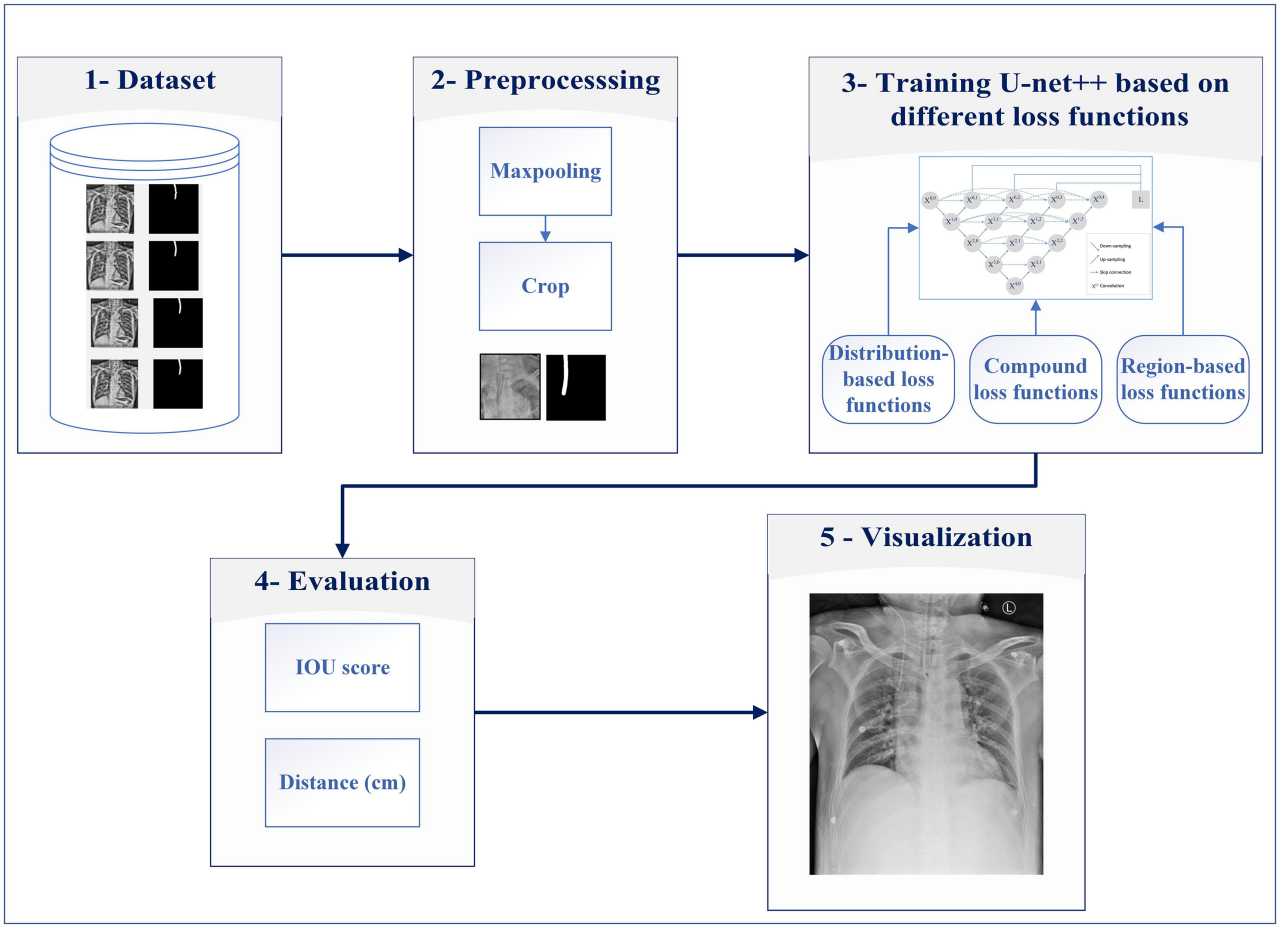
Proposed system for ETT segmentation.

The main contributions of this study are (1) evaluating different encoders, (2) investigating the different distribution (Dice, Tversky, and Jaccard) and region-based (BCE, Focal, and MCC) loss functions for training U-Net++, and (3) integrating distributed-based loss functions and region-based loss functions (compound loss functions) to obtain the best performance accuracy for ETT segmentation and localization, which is shown in Figure 1.

The proposed method consists of five major steps: dataset, preprocessing, developing a model based on different loss functions, evaluation, and visualization of the results.

Step 1: The data from the Dalin Tzu Chi Hospital are used for evaluating ETT segmentation.
Step 2: This step is preprocessing, which includes maxpooling and cropping for decreasing raw images' size (reducing computational complexity) and preparing them for the next step.
Step 3: In this step, the obtained images are fed into U-Net++ models for detecting ETT appropriately. In this step, distribution and region-based loss functions along with compound loss functions are used for ETT training.
Step 4: After obtaining the trained U-Net++ model, Euclidean distance between the predicted tip and the ground truth point are calculated, then the percentage of samples with the error less than a specific value (PSE) is obtained.
Step 5: Predicted tip and the ground truth point are shown on input images, in the visualization step.

It is worth mentioning that the procedure is carried out in three phases, namely training, validation, and testing. The segmentation task is performed only for the testing phase on unseen image data with different loss functions. The five steps of the proposed method are described as follows.

### Dataset

**Figure 2:**
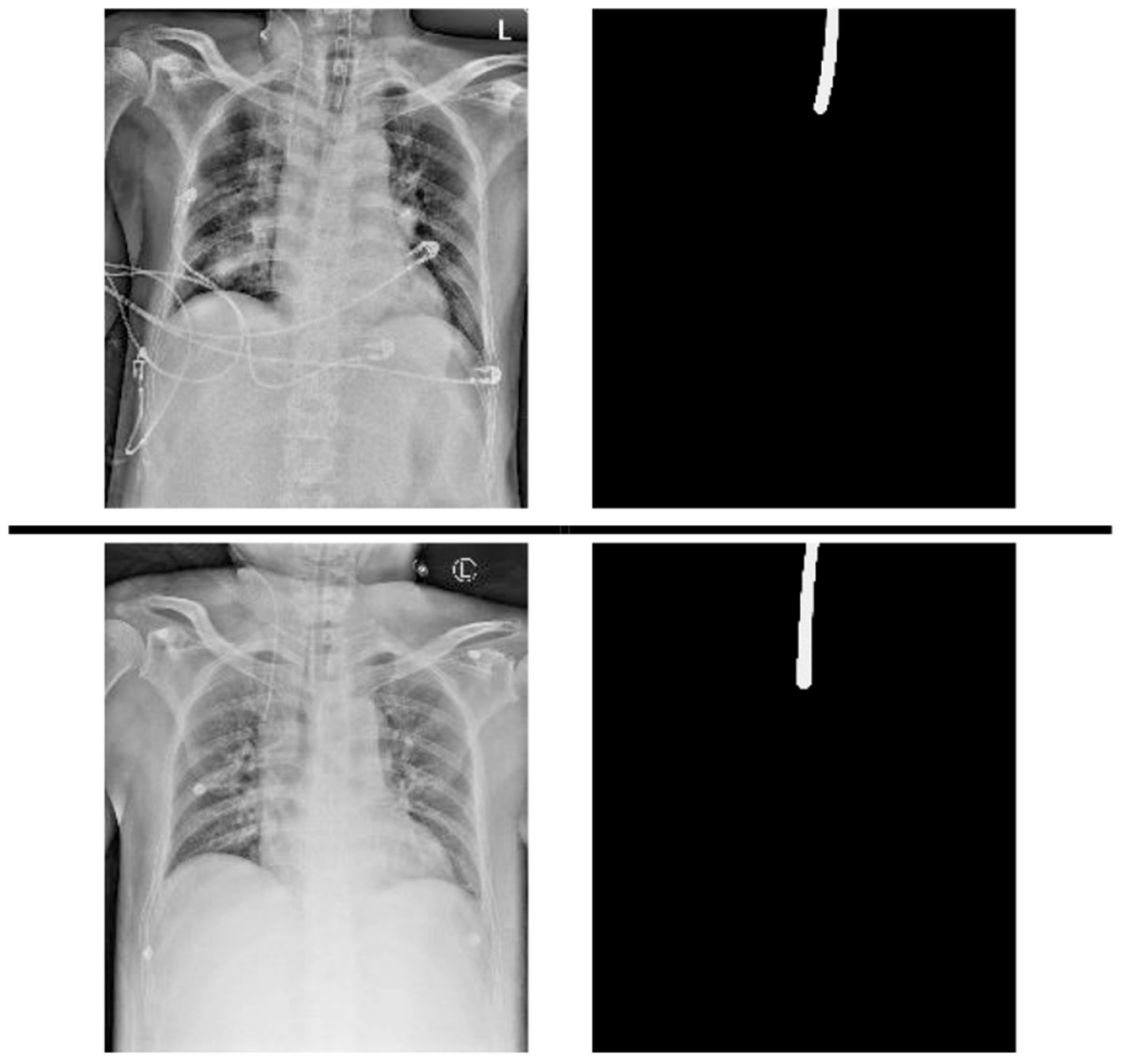
Samples of data and its annotation in the Dalin Tzu Chi dataset.

A chest x-ray dataset was applied to analyze the impact of the proposed method. The data were collected from chest radiographs of intubated ICU patients which had been confirmed by doctors from the Dalin Tzu Chi Hospital and who corrected the ground truth (see Figure 2).

The deviation of 5-fold cross validation is shown in Table 1, where 144 additional images from the Royal Australian and New Zealand College of Radiologists (RANZCR) Catheter and Line Position (CLiP) challenge dataset (Jarrel Seah et al., 2023) are also added to the training dataset of each fold. This study was approved by the institutional review board (IRB) of the Buddhist Dalin Tzu Chi Hospital (IRB number: B11103010).

**Table 1:** The amount of training, validation, and test samples in our dataset.

**Number of folds**	**Train (all data)**	**Validation**	**Test**
Fold 1	148 (292)	49	47
Fold 2	147 (291)	49	49
Fold 3	147 (291)	49	49
Fold 4	147 (291)	49	49
Fold 5	148 (292)	48	49

### Pre-processing

**Figure 3:**
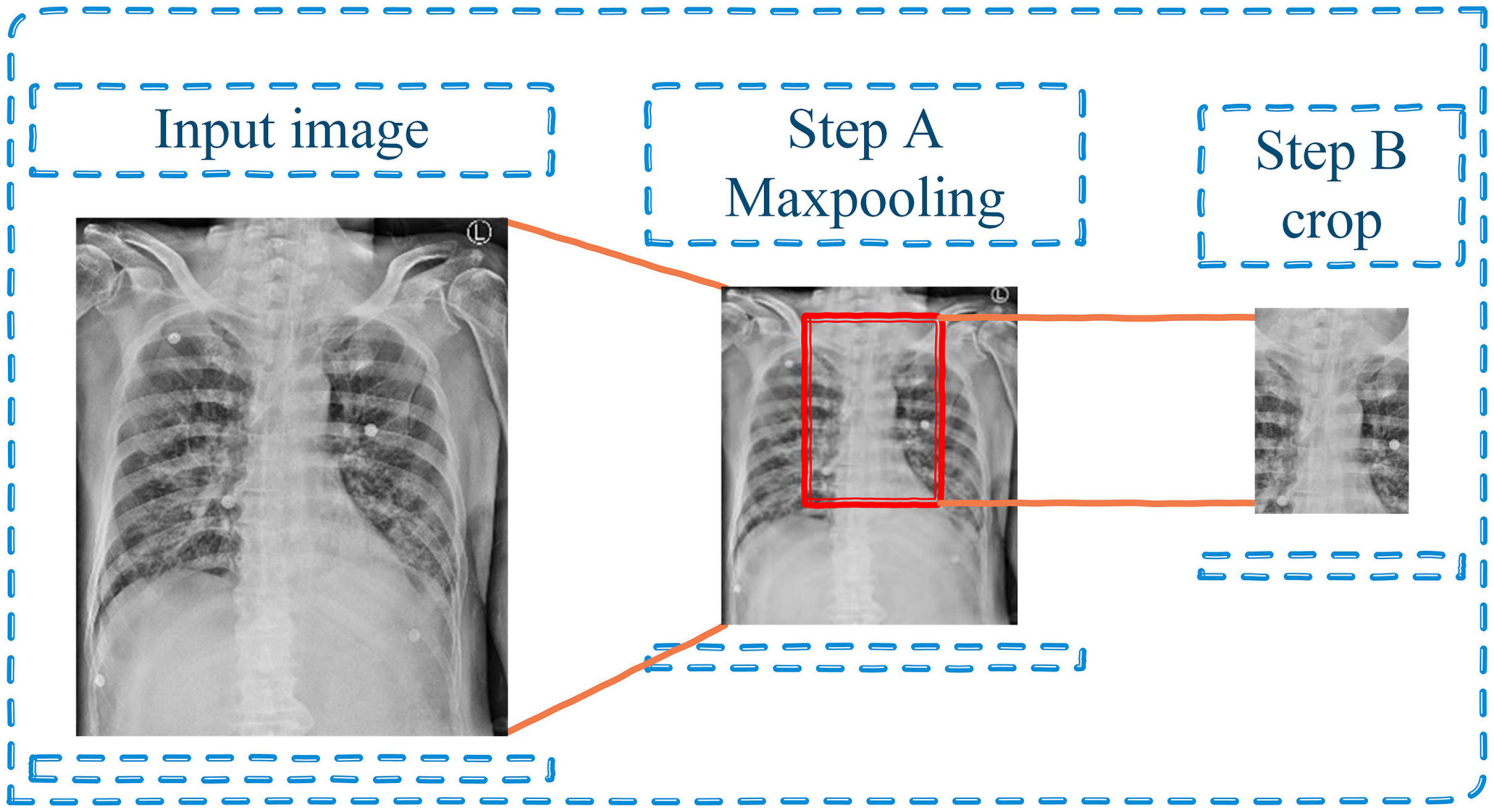
Preprocessing step—Maxpooling and cropping the input image.

For preprocessing, the input images were preprocessed to enhance the generalization ability and speed up the process of model training so that the model could be trained better. To downscale images by extracting the most important feature, the maxpooling with kernel 3 × 3 was performed (step A in Figure 3). Then, the images were cropped based on the center of images since this area is the approximate position of ETT (step B in Figure 3).

**Figure 4:**
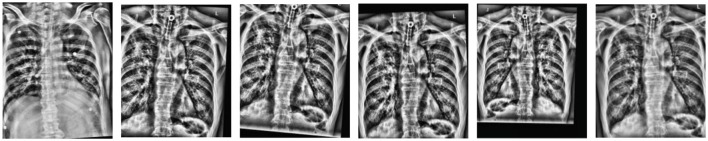
Samples of data augmentation (flip, affine, and rotation transforms).

Moreover, during the training stage, data augmentation techniques were used to reduce the overfitting and increase the robustness and generalizability of the model. First of all, 50% of input images were flipped horizontally around the y-axis. Then, affine transforms with scale range between 85 and 115% along with 5 degrees for rotation were randomly applied on these images, which then shifted for 0.1. Finally, Contrast Limited Adaptive Histogram Equalization (CLAHE) with an upper threshold value of (1, 4), and 0.7 for probability of applying the transform were applied. The augmentation stages were applied to both original input images and their ground-truth. Figure 4 displays several examples of data augmentation applied to the original input images.

### Deep learning model based on loss functions

**Figure 5:**
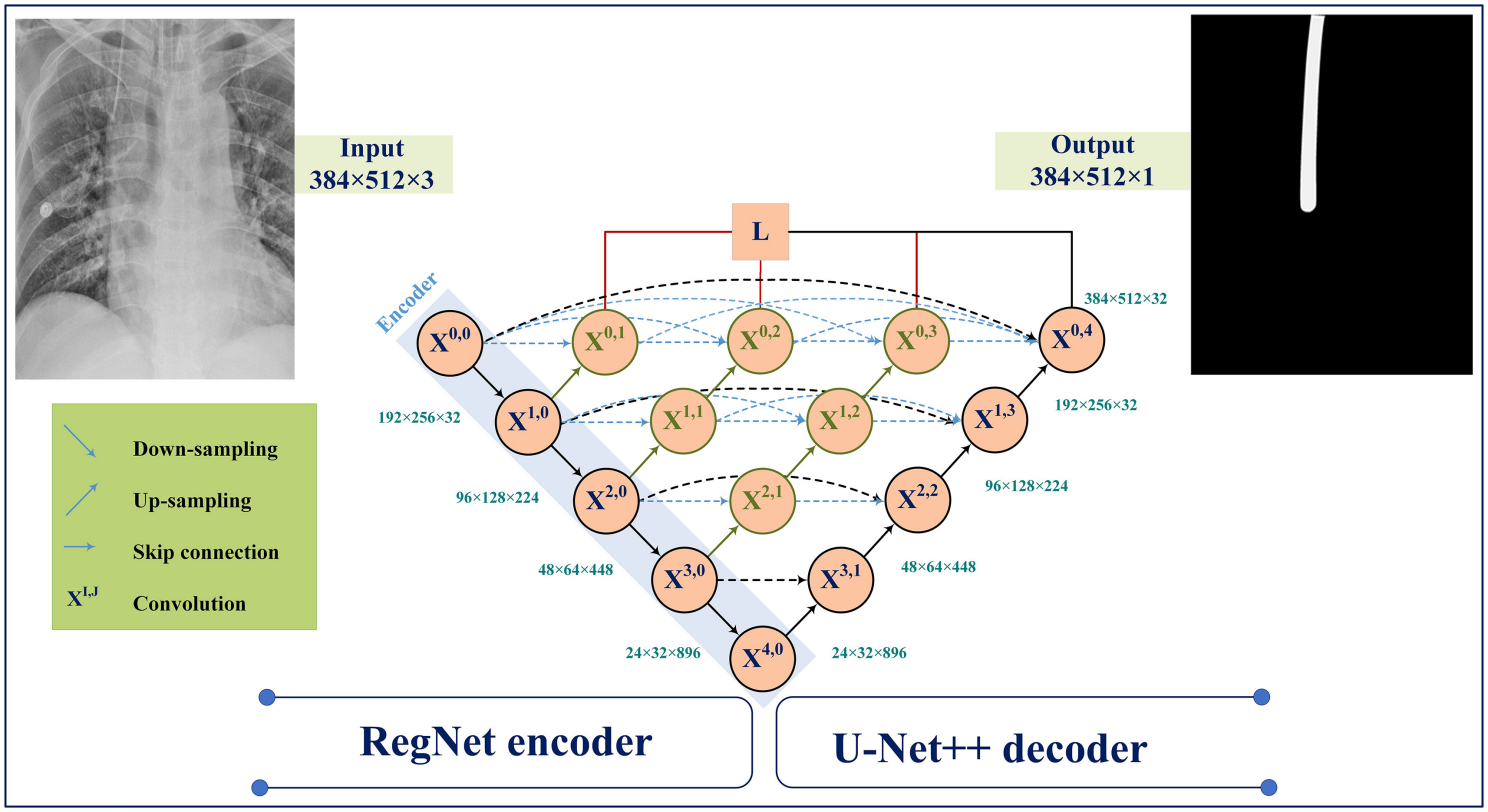
Model architecture using RegNet encoder with U-Net++ decoder.

The U-Net model was developed and applied to medical image segmentation, which submitted the best performance in two IEEE International Symposium on Biomedical Imaging (ISBI) challenges, including neural structure segmentation and cell tracking (Ronneberger et al., 2015). U-Net++ (Zhou et al., 2018) is the main architecture used in for ETT segmentation in this study. Figure 5 demonstrates the changes to the skip connection structure of the U-Net. The idea of improvement is to use the dense skip connections. Compared to the U-Net, these dense skip connections can maintain more detailed information by improving the encoder's feature maps before combining them with the decoders, whereas feature maps with the same scale can be combined through the U-Net.

Figure 5 illustrates the U-Net++ architecture utilized in our study. The main structure consists of an encoder sub-network followed by a decoder sub-network. The black sections indicate the original U-Net architecture, while the blue and green sections indicate the dense convolution blocks on the skip pathway. The red sections indicate the deep supervision mechanism. These components (red, green, and blue) are the primary differences between U-Net and U-Net++.

Suppose *x*^*i,j*^ denotes the output of *X*^*i,j*^ where *i* and *j* denote the down-sampling layer and the convolution layer. The *x*^*i,j*^ is formulated as follows:


(1) $$x^{i,j}=\left\{ \begin{array}{l} H\left(x^{i-1,j}\right)\;j=0\\ \;H\left(\left[{\left[x^{i,k}\right]}_{k=0}^{j-1},\;\prod (x^{i+1,j-1})\right]\right)\;j>0 \end{array}\right.$$

Here, ∏(.) indicates the up-sampling layer and H(.) denotes a convolution operation. Also, the notation [ ] represents the concatenation layer.

ResNet and RegNet have an acceptable performance in the recent deep learning applications (He et al., 2016; Radosavovic et al., 2020). Hence, we have explored the use of ResNet and RegNet on ImageNet as an encoder for feature extraction.

**Figure 6:**
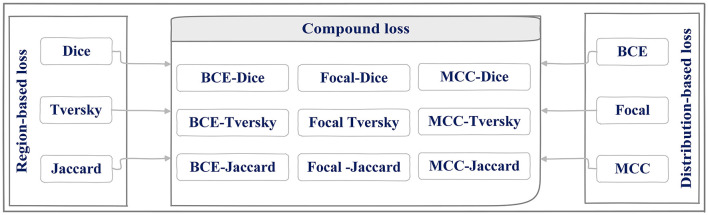
Region, distributed loss functions, and different combination of them (compound) used for U-Net++ training phase.

U-Net++ model has demonstrated a high performance in the previous ETT segmentation works (Frid-Adar et al., 2019). In this paper, distributed, region, and compound loss functions, which are used for training the U-Net++ model, can be listed as Binary Cross Entropy (BCE) loss (Kuang and Tie, 2021), Dice loss (Shen et al., 2018), Focal loss (Li et al., 2020), Jaccard loss (Duque-Arias et al., 2021), Tversky loss (Salehi et al., 2017), and MCC (Chen et al., 2016; shown in Figure 6).

Loss functions that have been applied in the current approach are going to be reviewed in this section briefly. Before that, however, it is worth clarifying the definitions of these three categories and their natures.

As shown in Figure 6, three different loss functions are evaluated and proposed in this paper. The first category, distributed-based loss functions, are based on the distribution of labels (Rajaraman et al., 2021) whereas Region-based loss functions have been applied to decrease the mismatch or increase the overlap regions among ground truth and predicted value in segmentation (Jadon, 2020). Each loss function is going to be introduced here.

### Distribution-base loss function

#### BCE loss

Binary cross-entropy loss function (Kuang and Tie, 2021) has been implemented for classification problems with a single output unit whereas categorical cross-entropy can be considered as the loss function for multiclass classification. This loss function combines a sigmoid layer with a BCE Loss in one single class. The presented version is numerically stable in comparison with previous plain sigmoid along with a BCE loss function implemented on one layer which has taken advantage of applying the long-sum-exp trick. If I unreduced (i.e., with reduction set “o ‘n' ne”) loss between ground truth (*y*) and (*x*) stand as predicted value then loss can be described as:


(2) $$\begin{array}{l} l\left(x,y\right)=BCEL={\left\{l_{1,\ldots ,}l_{N}\right\}}^{T}\\ l_{n}=\;-\omega_{n}\left[y_{n}.log\sigma \left(x_{n}\right)+\left(1-y_{n}\right).\log\left(1-\sigma \left(x_{n}\right)\right)\right] \end{array}$$

Where *l* stands for values of loss in different batches, *BCEL* is the set of loss values, *N* indicates the batch size, *σ* is the sigmoid function, and *ω* is the weight of *n*th input. If the value of reduction is not equal to “none” (default “mean”) then:


(3) $$l\left(x,y\right)=\;\left\{ \begin{array}{l} mean\left(L\right),\;if\;reduction=‘mean’;\;\\ sum\left(L\right),\;if\;reduction=‘sum’ \end{array}\right.$$

It is used to measure the rate's error of a reduction, for instance an auto-encoder. Remember that the ground truth *y*[*i*] should depict its values in the interval of [0,1].

By adding weights to positive instances there would be the possibility of commutating recall and precision. In the case of multi-label classification, the given formula can be used to describe the loss function:


(4) $$\begin{array}{l} l_{c}\left(x,y\right)=L_{c}={\left\{l_{1,c},\ldots ,l_{N,c}\right\}}^{T}\\ L_{n,c}=-\omega_{n,c}\left[p_{c}y_{n,c}.log\sigma \left(x_{n,c}\right)+\left(1-y_{n,c}\right).\log\left(1-\sigma \left(x_{n,c}\right)\right)\right] \end{array}$$

Where *c* is the class label (*c* > 1 for multi-label binary classification, *c* = 1 for single-label binary classification), n is the sample's number in each single batch, and *p*_*c*_ stands as the weight of the positive answers for the class c. If *p*_*c*_ > 1 then it is expected to have an increase in the recall and in case of *p*_*c*_ < 1 the precision's value would increase.

#### Focal loss

Class imbalance has been addressed through focal loss (Li et al., 2020) during training steps for problems like object detection. By implementing a modulating term to the cross-entropy, focal loss focuses on learning the hard-misclassified instances. It can be considered as a dynamically scaled cross entropy loss function, where the scaling factor goes to zero as the confidence in the correct class increases in value. This scaling factor would automatically decrease the weight of easy samples' contribution during the training process while simultaneously causing the model to focus on hard ones. Generally, the factor ${\left(1-p_{t}\right)}^{\gamma }$ has been added to the standard cross entropy criterion throughout the focal loss function. It should be mentioned that *p* is a value between 0 and 1 which is known as the model's estimated probability and *p*_*t*_ is defined as


(5) $$p_{t}=\{\begin{array}{c} p\;if\;y\left(label\;of\;sample\right)=1\\ \;1-p\;O.W \end{array}$$

Setting γ > 0, which is known as tunable focusing parameter, would reduce the relative loss for well-classified samples (*p*_*t*_ > 0.5), and would attract more attention on hard, misclassified samples. Here, the tunable *focusing* parameter is a non-negative value (γ ≥ 0).


(6) $$\begin{array}{l} FL\left(p_{t}\right)=\;{-\left(1-p_{t}\right)}^{\gamma }\log\left(p_{t}\right) \end{array}$$

#### MCC loss

MCC loss (Chen et al., 2016) is an informative metric which can also be implemented in cases of skewed distributions and has been shown to be an optimal metric when designing classifiers for imbalanced classes. The most useful expression for computation was from the original article by Gorodkin. Multi-class MCC is often called “*R*_*K*_ statistics.”


(7) $$\begin{array}{l} R_{K}=\frac{NTr\left(C\right)-\sum_{kl}{\tilde{C}}_{K\;}{Ĉ}_{l}}{\sqrt{N^{2}-\sum_{kl}{\tilde{C}}_{k}{{Ĉ}_{l}}^{T}}\;\sqrt{N^{2}-\sum_{kl}{{\tilde{C}}_{k}}^{T}{Ĉ}_{l}}} \end{array}$$

Where N is the samples' number, ${\tilde{C}}_{k}\;$is the *Kth* row of the confusion matrix *C*, ${\tilde{C}}_{l}\;$the 1th column of *C*, *C*^*T*^ is *C* transposed, and *Tr*(*C*) is the trace of *C*.

### Region-based loss function

#### Dice loss

Dice loss (Shen et al., 2018) is a metric function used to evaluate the similarity of two samples in the given interval of [0,1]. The larger the value, the more similar the two mentioned samples are.


(8) $$\begin{array}{l} DL\left(y,x\right)=1-\frac{2yx+1}{y+x} \end{array}$$

Where *x* is the prediction of the model and *y* is its ground truth value.

#### Jaccard loss

Jaccard loss (Duque-Arias et al., 2021) and Dice loss are similar; it is also applied to optimize the segmentation metric directly. It actually calculates the similarity between two finite sample sets (ground truth values and predicted values), and it is defined as the measure of the mentioned set's intersection divided by the measure of their union:


(9) $$\begin{array}{l} J\left(x,y\right)=\frac{\left|x\cap y\right|}{\left|x\cup y\right|} \end{array}$$

#### Tversky loss

Tversky loss assigns different weights to false negative (FN) and false positive (FP). This is different to dice loss as it uses equal weights for FN and FP (Salehi et al., 2017).

Let *α*, *β* > 0 and *y, x* ∈ [0, 1]^*M*^. Then the smooth Tversky index corresponding to *y* and *x* respectively defined as ground truth and the output of the predicted value is defined as:


(10) $$\begin{array}{l} \tau_{\alpha ,\beta }\left(y,x\right)=\frac{yx}{yx+\;\alpha \left(1-y\right)x+\beta y\left(1-x\right)} \end{array}$$

Clearly if *y, x* ∈ {0, 1}^*M*^, τ_*α*,*β*_ (*y, x*)then it is the same as the discrete Tversky index described by the given equation:


(11) $$\begin{array}{l} T_{\alpha ,\beta }\left(y,x\right)=\frac{TP}{TP+\alpha FP+\;\beta FN} \end{array}$$

In the given formula, TP stands for True Positive and FP and FN are known as false positives and false negatives, respectively.

### Compound loss function

Getting close to the ground truth value of the ETT position motivated the authors to implement and evaluate different loss functions from distribution and region-based loss function and combine them to find the best loss function for U-Net++ training phase. Doing so would eliminate the mentioned obstacles that might happen in hospitals and reduce the risks of ETT mispositioning, thereby improving the safety of the procedure. Nine different combinations of distribution-based and region-based loss functions (compound loss part in Figure 6) that have been studied in the presented paper are going to be discussed in the further section of the paper.

### Evaluation metrics and visualization

Two performance evaluations are considered for the effectiveness of the U-Net++ model with different backbones and different loss functions: IOU [Jaccard loss in Equation (9)] for evaluating ETT segmentation and calculating distance between real ground truth and predicted ETT for assessment of the ETT localization.

**Figure 7:**
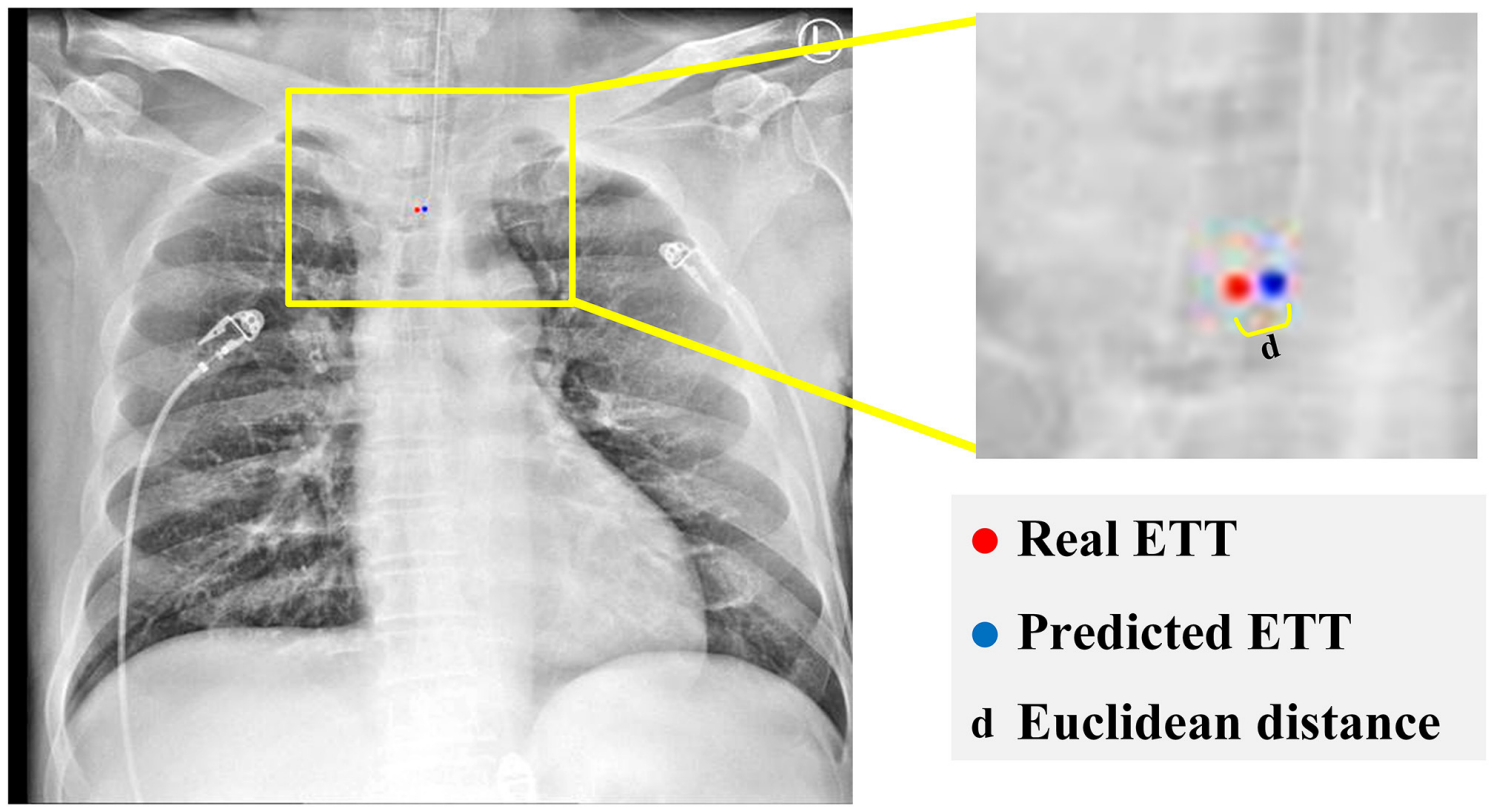
Calculate the distance between real and predicted ETT.

Figure 7 represents the Euclidean distance between the predicted tip and the ground truth point is calculated through:


(12) $$d=\sqrt{{\left(b_{2}-b_{1}\right)}^{2}+{\left((a_{2}-a_{1}\right)}^{2}}$$

Where (*a*_1_, *b*_1_) and (*a*_2_, *b*_2_) are respectively considered as the coordinates of the predicted tip and the ground truth tip positions.

The percentage of samples with the error less than a specific value (PSE) is given by:


(13) $$\begin{array}{l} PSE=\frac{Number\;of\;samples\;with\;d<value}{Total\;number\;of\;samples}\times 100 \end{array}$$

Where value belongs to {0.25, 0.5, 1, 1.5, 2} and *d* stand for calculated Euclidean distance for each sample.

## Results

The different experiments on the dataset (described in Section 3.1) are conducted to evaluate the impact of loss functions for ETT segmentation and the effectiveness of the proposed method. The results for different loss functions and different encoders are presented and compared. Finally, the distance between the true location and predicted location obtained from the proposed method are compared. All simulations have been carried out in Python 3.8.13 using PyTorch package on a machine equipped with core i9 process with speed 2.90 GHz and 64 GB of memory and NVIDIA GeForce RTX 3080 Ti.

### Evaluation of different encoders

The main step in image processing is feature extraction, which was provided by statistical algorithms or applying some filters in the past. However, features have recently been extracted automatically by introducing deep learning models. Some have become popular and applied for different data analysis domains. These networks are now used for features extraction or in the beginning of any deep learning model and its named encoders. In this paper, the three encoders are evaluated as the pretrained neural network: regnetX_120, regnetY_120 from RegNet encoder, and resnet50 from ResNet encoder. The IOU scores are calculated for these encoders and presented in Table 2.

**Table 2:** IOU score obtained from UNet++ for different encoders.

**Encoders**	**IOU score**					
	**Fold1**	**Fold2**	**Fold3**	**Fold4**	**Fold5**	**Mean**
regnetX_120	0.8757	0.8554	0.7916	0.8453	0.8711	0.8478
regnetY_120	0.9033	0.8253	0.7260	0.8036	0.8331	0.8182
resnet50	0.8796	0.8558	0.7237	0.8036	0.8331	0.8191

As is expected, the IOU score which has been applied to object detection problems can measure the overlaps of a predicted value vs. actual bounding box of an object. The closer the predicted bounding box values to their actual bounding box values, the bigger the intersection would be and as a result the value of IOU would increase (Kim and Lee, 2020).

In Table 2, obtained values of IOU Score related to three different implemented encoders, namely regnetX_120, regnetY_120, and resnet50, on five various folds along with their means are summarized. Thus, according to the definition of IOU criteria, performance of regnetX_120 on Fold1 has been allocated the IOU value of 0.8757 in comparison with other encoders. Performance of regnetY_120 model on the 1st-fold has allocated the maximum value of 0.9033 among the other encoders and the same situation happened for resnet50 with the maximum value of 0.8796. According to the presented mean values of the models, it can be concluded regnetX_120 with the mean value of 0.8478 has shown the best performance in predicting the ETT position among the mentioned encoders.

### Evaluation of different loss function

The calculated IOU scores on the training dataset for different loss functions are listed in Table 3 along with their mean values. From the presented values, it can be seen that the maximum IOU score related to BCE loss function happened on Fold1 with a value of 0.8648 but, by considering Dice loss function as a substitute of the previous loss function, its related IOU score would still occur on the same encoder, namely Fold1 with the value of 0.8583. For the remaining loss functions, namely Focal, Jaccard, and MCC loss functions, the IOU score has allocated its maximum values on Fold1 respectively as 0.8849, 08571, and 08841. In the case of Tversky loss function, the IOU score achieved its highest value on Fold 5 with the value of 0.8396. Based on the obtained mean values listed in the given table, MCC loss function would lead to the highest value of IOU score which can be seen as the best implemented loss function. In terms of IOU score, distribution-based loss functions performed better than region-based loss functions. The average value corresponds to the mean parameter for mentioned loss functions respectively obtained as 0.8361 and 0.7560.

**Table 3:** IOU score for ETT segmentation based on single loss function.

**Groups**	**Loss function**	**IOU score**					
		**Fold1**	**Fold2**	**Fold3**	**Fold4**	**Fold5**	**Mean**
Region-base loss	Dice	0.8583	0.8453	0.7290	0.7391	0.7797	0.7902
	Tversky	0.6864	0.7201	0.7119	0.7541	0.8396	0.7424
	Jaccard	0.8571	0.7007	0.6322	0.7229	0.7653	0.7356
						Average =	0.7560
Distribution-based loss	BCE	0.8648	0.8377	0.7893	0.8421	0.7865	0.8240
	Focal	0.8849	0.8319	0.8004	0.8308	0.8412	0.8378
	MCC	0.8841	0.8419	0.7732	0.8731	0.8616	0.8467
						Average =	0.8361

### Evaluation ETT segmentation for combining loss functions

IOU score values of hybrid loss functions are listed in Table 4. These functions are BCE-Dice, BCE-Jaccard, BCE-Tversky, Focal-Dice, Focal-Jaccard, Focal-Tversky, MCC-Dice, MCC-Tversky, and MCC-Jaccard. The maximum value of IOU score in Fold 1 is related to MCC-Dice loss function with the value of 0.9005. For Fold 2, the mentioned value is related to the BCE-Dice loss function with the value of 0.8839, whereas IOU has allocated the maximum value on Fold 3 through Focal-Dice with the value of 0.8283. In the case of Fold 4, IOU reached its maximum value of 0.8725 through MCC-Jaccard loss function. BCE-Dice loss function has assigned the maximum value of 0.8852 to IOU parameter on Fold 5. The mean value of each loss function is given in the last column of the table and is based on the achieved maximum value which is related to the MCC-Tversky loss function; it has the best performance among all nine loss functions on all 5-folds. Moreover, the average value of IOU score for all combinations is 0.8601, which demonstrates the better performance of individual region-based and distribution loss functions (in Table 3). Therefore, the integration of the region and distribution-based loss functions demonstrated the higher capability for ETT segmentation based on the achieved IOU score.

**Table 4:** IOU score for ETT segmentation based on combined loss functions.

	**Loss function**	**IOU score**					
		**Fold1**	**Fold2**	**Fold3**	**Fold4**	**Fold5**	**Mean**
1	BCE-Dice	0.8963	**0.8839**	0.7691	0.8603	**0.8852**	0.8590
2	BCE-Jaccard	0.8965	0.8498	0.7915	0.8671	0.8728	0.8555
3	BCE-Tversky	0.8697	0.8712	0.7781	0.8707	0.8728	0.8525
4	Focal-Dice	0.8963	0.8617	**0.8283**	0.8709	0.8689	0.8652
5	Focal-Jaccard	0.8928	0.8623	0.8175	0.8691	0.8800	0.8643
6	Focal-Tversky	0.8934	0.8802	0.8128	0.8706	0.8733	0.8661
7	MCC-Dice	**0.9005**	0.8550	0.7801	0.8687	0.8667	0.8542
8	MCC-Tversky	0.8984	0.8775	0.8247	0.8711	0.8699	**0.8683**
9	MCC-Jaccard	0.8812	0.8753	0.7932	**0.8725**	0.8594	0.8563
						Average =	0.8601

The bold values in each column indicates the highest IOU score.

### Distance between real and prediction ETT locations

In Table 5, the distance between real and prediction ETT locations are summarized. Minimum distance obtained through distribution-based loss functions including BCE, Focal and MCC is related to Focal loss function whereas the Dice loss function has shown the best performance among the other region base loss functions to determine the average distance on 5 studied folds. The maximum value of mean parameters on all 5 studied folds for the entire set of loss functions is related to the MCC-Tversky with the value of 0.8683, which demonstrates its great performance on different folds. In the third group of the given table, which is related to the compound loss functions and includes BCE-Dice, BCE-Jaccard, BCE-Tversky, Focal-Dice, Focal-Jaccard, Focal-Tversky, MCC-Dice, MCC-Tversky, and MCC-Jaccard, the best fitted value is related to the first member of this group with the value of 0.2901. Since obtaining the minimum error between ground truth value and model prediction of ETT position is the main objective of the given table, BCE-Dice loss function from the compound loss function group has the best performance among all implemented loss functions with a mean value of 0.2901. Three different values have been calculated correspond to three different groups of loss functions. Values of the mean parameter corresponding to distribution-based loss, region-based loss, and compound loss functions have been obtained as 0.3878, 0.6096, and 0.3348. Therefore, the proposed compound loss functions have shown the best performance for ETT localization among the studied groups, whereas in the other two cases the distribution-based loss functions have better performance for ETT localization.

**Table 5:** The calculated distance (cm) between real and predicted ETT locations.

**Group**	**Loss function**	**Distance (cm)**					
		**Fold1**	**Fold2**	**Fold3**	**Fold4**	**Fold5**	**Mean**
Region-based loss	Dice	0.3244	0.3741	0.6841	0.6814	0.5624	0.5253
	Tversky	0.7420	0.4166	0.5843	0.5407	0.4392	0.5446
	Jaccard	0.3909	0.5124	1.6230	0.7222	0.5468	0.7591
						Average =	0.6096
Distribution-based loss	BCE	0.3847	0.4069	0.4670	0.3993	0.3498	0.4015
	Focal	0.2225	0.4317	0.3255	0.4192	0.4765	0.3751
	MCC	0.2996	0.4503	0.4447	0.3289	0.4114	0.3870
						Average =	0.3878
Compound loss	BCE-Dice	0.1835	0.2579	0.3905	0.3385	0.2803	**0.2901**
	BCE-Jaccard	0.1728	0.4875	0.3546	0.3446	0.3897	0.3498
	BCE-Tversky	0.2464	0.3244	0.4573	0.3880	0.3897	0.3611
	Focal-Dice	0.2180	0.5053	0.2742	0.3420	0.3489	0.3377
	Focal-Jaccard	0.2154	0.3368	0.3352	0.3454	0.2734	0.3012
	Focal-Tversky	0.2304	0.2455	0.3052	0.2899	0.4418	0.3168
	MCC-Dice	0.1959	0.3377	0.4748	0.3506	0.4079	0.3534
	MCC-Tversky	0.2358	0.3014	0.4137	0.4270	0.4019	0.3560
	MCC-Jaccard	0.3608	0.2420	0.3449	0.3897	0.3993	0.3473
						Average =	0.3348

The bold value indicates the minimum average error on different studied folds.

An ideal ETT placement has been measured by the distance between ETT's tip and its ground truth value. Five different categories based on misplacement of ETT have been shown in Table 6. The first category which has been indicated by Error <0.25(cm) depicts the percentage of error's rate in ETT placement among 15 different studied loss functions which has been studied on test samples. It can be seen that BCE_Jaccard loss function has achieved the best performance in ETT placement, since 65.47% of test samples have errors <0.25 between model prediction and its ground truth value. The same measurements in the cases of the other four categories have been investigated. BCE_Jaccard loss function in 88.81% of studied test cases has depicted error values <0.5; in 95.29% of studied test samples Focal_Tversky loss function has error values <1; in 97.82% of studied test samples Focal_Tversky loss function has shown errors with values of <1.5; and 98.32% of studied test samples which have implemented Focal_Dice loss functions have error values of <2.

**Table 6:** Comparison of distribution, region, and compound loss function for ETT localization.

**Group**	**Loss function**	**PSE (%)**				
		**Error**<**0.25**	**Error**<**0.5**	**Error**<**1**	**Error**<**1.5**	**Error**<**2**
Distribution-based loss	BCE	42.77	74.56	93.99	96.55	97.39
	Focal	53.92	84.11	92.74	95.34	96.17
	MCC	53.13	83.16	93.97	94.39	96.08
Region-based loss	Dice	45.27	77.41	91.06	94.00	96.14
	Tversky	46.38	69.48	84.45	90.55	93.53
	Jaccard	31.84	59.57	80.48	86.97	90.46
Compound loss	BCE_Dice	62.08	88.77	94.41	96.93	97.39
	BCE_Jaccard	**65.47**	**88.81**	93.14	95.71	97.86
	BCE_Tversky	59.44	84.44	92.25	94.40	96.50
	Focal_Dice	61.65	87.14	94.87	97.43	**98.32**
	Focal_Jaccard	63.91	87.08	94.44	96.14	97.86
	Focal_Tversky	63.58	87.99	**95.29**	**97.82**	98.28
	MCC_Dice	59.34	82.57	93.91	96.14	97.44
	MCC_Tversky	61.68	87.56	93.57	95.67	96.55
	MCC_Jaccard	63.80	85.32	93.13	95.76	96.18

The bold values in each column indicate the highest PSE performance through its related loss function.

## Conclusion

In this paper, the impact of different loss functions and encoders is evaluated for ETT segmentation and localization. Moreover, the various combinations of region-based and distributed-based loss functions were assessed to obtain the optimum configuration of U-Net++ for ETT segmentation. We tested the proposed integration loss functions for training U-Net++ on CXR from the Dalin Tzu Chi Hospital database. The results and comparison analyses demonstrate the robustness of segmentation and localization performance of the presented technique. According to the obtained results, the correspondent average IOU scores for ETT segmentation related to distribution, region, and compound loss function groups respectively are given as 0.8361, 0.7560, and 0.8601. The best IOU score is obtained by integration of MCC and Tversky (MCC-Tversky from the proposed compound group) with the value of 0.8683. Based on the calculated distance between the real and predicted position of ETT, the compound groups demonstrate the better performance for ETT localization. The minimum error was obtained through BCE-Dice with the value of 0.2901 cm from the proposed compound loss function groups.

In future works, we plan to pursue four significant study streams. Firstly, we will consider different methods of combining loss functions to improve the performance of ETT segmentation, including weighting schemes. Secondly, we will investigate and extend the proposed compound loss functions to a more generic framework for ETT and Carina segmentation and localization. Thirdly, we will optimize the proposed method for real-time applications. Finally, we will explore the development of a new deep learning model to further improve segmentation accuracy.

## Data availability statement

The raw data supporting the conclusions of this article will be made available by the authors, without undue reservation.

## Ethics statement

This study was approved by the institutional review board (IRB) of the Buddhist Dalin Tzu Chi Hospital (IRB Number: B11103010). Written informed consent for participation was not required for this study in accordance with the national legislation and the institutional requirements.

## Author contributions

C-CH: conceptualization, resources, funding acquisition, formal analysis, supervision, and project administration. RA: software, methodology, and writing—original draft preparation. C-WL and J-SH: data curation, resources, and review and editing. MB: methodology and writing—review and editing. AY: writing—review and editing and visualization. SB: conceptualization, writing—review and editing, and supervision. T-KL: data curation, resources, and writing—review and editing. W-LF: data curation, resources, writing—review and editing, and funding acquisition. All authors contributed to the article and approved the submitted version.
